# A systematic review of observational practice for adaptation of reaching movements

**DOI:** 10.1038/s41539-024-00271-5

**Published:** 2024-10-03

**Authors:** Julian Rudisch, Luis K. H. Holzhauer, Karmen Kravanja, Fred H. Hamker, Claudia Voelcker-Rehage

**Affiliations:** 1 Department of Neuromotor Behavior and Exercise, Institute of Sport and Exercise Sciences, University of Münster, Münster, Germany; 2 Department of Sports Analytics, Institute for Sport Science, Saarland University, Saarbrücken, Germany; 3 Department of Psychology, Faculty of Mathematics, Natural Sciences and Information Technologies, University of Primorska, Koper, Slovenia; 4 Department of Computer Science, Chemnitz University of Technology, Chemnitz, Germany

**Keywords:** Cognitive neuroscience, Human behaviour

## Abstract

Observational practice is discussed as a substitute for physical practice for motor learning and adaptation. We systematically reviewed the literature on observational practice in reaching and aiming tasks. Our objectives were to identify (i) performance differences between observational and physical practice; (ii) factors that contribute to adaptation following observational practice; and (iii) the neural correlates of observational practice. We found 18 studies, all investigated adaptation of reaching in visuomotor rotations or force-field perturbations. Results of the studies showed that observational practice led to adaptation in both, visuomotor rotation and force-field paradigms (*d* = −2.16 as compared to no practice). However, direct effects were considerably smaller as compared to physical practice (*d* = 4.38) and aftereffects were absent, suggesting that observational practice informed inverse, but not forward modes. Contrarily, neurophysiological evidence in this review showed that observational and physical practice involved similar brain regions.

## Introduction

Motor learning—as it is defined in this paper—encompasses the acquisition of new unknown skills or tasks as well as the improvement or adaptation of skills acquired in the past. While movement skills are typically learned through repeated task execution (i.e., physical practice), observation of a model or another actor executing the task (i.e., observational practice), can have beneficial effects for the acquisition of new skills and adaptation of previously learned skills. Some evidence exists about the benefits, modalities, and potential underlying brain mechanisms when learning motor skills from observational practice, (i.e., observational learning) in upper extremity tasks, such as reaching^1^. Reaching, aiming, and grasping are involved in many (instrumental) activities of daily living and frequently targeted following motor impairments such as stroke. Further, reaching plays a prominent role in the interaction with technical devices and human-like robots (or robotic arms) might learn reaching actions in a similar fashion to humans. Observational practice can supplement or enhance the learning of reaching skills through physical practice^2^. In the context of sports sciences research, observational learning of more complex upper extremity skills, such as juggling, or a tennis serve has been intensively investigated in the past^3^. However, to date, no systematic review of the literature on observational learning of relatively simple reaching, aiming, and grasping tasks that are relevant for activities of daily living has been conducted. In this study, we therefore systematically reviewed studies on observational practice in reaching, aiming, and grasping tasks. Our aim was, to investigate whether observational practice of upper extremity reaching movements leads to similar learning rates and involves similar neural (brain) mechanisms as compared to physical practice and whether there are factors that determine learning success.

It has been well established that we do not only learn and adapt motor skills through physical, but also through mental practice. This includes the imagination of oneself performing the action (i.e., motor imagery) as well as the observation of another person (i.e., action observation) performing the same action. The potential of learning from observing a demonstrator (i.e., observational practice) as a substitute or complementary mode of practice is discussed to be particularly useful in situations where novel complex movements are learned, e.g., for sports or playing musical instruments^4^, when movement execution is restricted, e.g., in rehabilitation^5^ or when unskilled practice may have severe consequences and prior development of expertise is crucial, e.g., when performing surgery^6^. Whether learning from observing others can complement or even replace physical practice depends on different factors, such as the complexity of the task, the current skill level as well as its attentional and motivational state of the learner^7^.

Research on the effects and mechanism of observational practice dates back to the work of Bandura and colleagues on the social learning theory^8^, where learners observe and subsequently apply social behaviour (such as aggressive behaviour towards a doll). Their research suggests that observed behaviour is translated into a symbolic memory code and stored in form of a cognitive memory representation. This requires cognitive processes such as attention, retention, reproduction, and motivation. With the discovery of mirror neurons^9^, a physiological explanation for observational learning was introduced^10,11^. Mirror neurons have been described as a special set of neurons in the ventral premotor area (F5) of the macaque monkey that exhibit the same discharge behaviour when performing an action and when observing another primate performing the same action^12^. While a direct proof of mirror neurons (i.e., on a cellular level) in the human brain is lacking to date, evidence exists from neuroimaging studies that several networks in the brain show mirror-like behaviour^10^. Extensive neuroimaging research has been performed in the past to unravel the mirror-neuron system in humans i.e., an extended brain network that displays functional activity when observing an action performed by another actor. Meta-analyses of brain imaging studies have demonstrated mirror-like properties for a widespread network of clusters distributed across the brain^13–15^. These include, for example, the inferior frontal gyrus, the dorsal and ventral premotor cortex, but also sensory cortices and the cerebellum. Consequently, observational practice, may lead to similar processes as physical practice on a brain level, namely, to the modification and consolidation of task-specific sensorimotor networks^16,17^.

Many tasks and approaches have been used to investigate observational motor learning. Tasks differ with respect to the skill knowledge (learning of unknown skills versus practice of known skills), the expertise level of the learner (novices versus experts), and the type of task (upper versus lower extremity tasks). Ramsey, et al. ^1^ differentiate between two broad types of tasks: sequence learning and motor adaptation. Sequence learning comprises tasks that follow a sequential structure such as playing the piano, riding a bike, or dancing. Motor adaption is the process of gradually adjusting a pre-existing skill to novel constraints based on error feedback^18^ that leads to the acquisition or recallibration of motor skills^19–21^. Consequently, the specific skill (e.g., throwing) keeps its identity, but certain parameters such as the generated forces change due to changing constraints (e.g., weight of the thrown object or a force field). It is important to note that both, sequence learning as well as motor adaptation can occur on different time-scales. Lohse, et al. ^22^ define three distinct time-scales: Short-term motor learning typically refers to practicing a task less than an hour and corresponds to the initial fast learning phase^23^, medium-term refers to one hour to less than 24 h of practice, and long-term practice referring to more than 24 h to five weeks of practice.

From a motor control point of view, motor adaptation is achieved through updating the inverse model (which estimates the necessary motor commands for an action), and/or the forward model (which incorporates the expected sensory consequences of an action for rapid feedback control). A signature of a successful adaptation are so-called aftereffects^24^. That is, when being exposed to the original null-field immediately after having adapted to a force-field, the sensorimotor control system produces errors and requires time to re-adapt to the original constraints^19^. In the following we will focus on motor adaptation in reaching and aiming tasks as most activities of daily life require the maintenance of consistent performance in response to bodily (such as fatigue) or environmental changes (such as perturbations).

We systematically reviewed the literature on observational learning in reaching and aiming with special focus on force-field learning and visuomotor adaptation tasks. Our objectives were to investigate (i) if there are differences in adaptation related outcomes between observational and physical practice; (ii) what factors contribute to successful observational learning; and (iii) what the neurophysiological underpinnings of observational learning are and how information is processed and stored through observational learning.

## Results

### Study selection

**Fig. 1:**
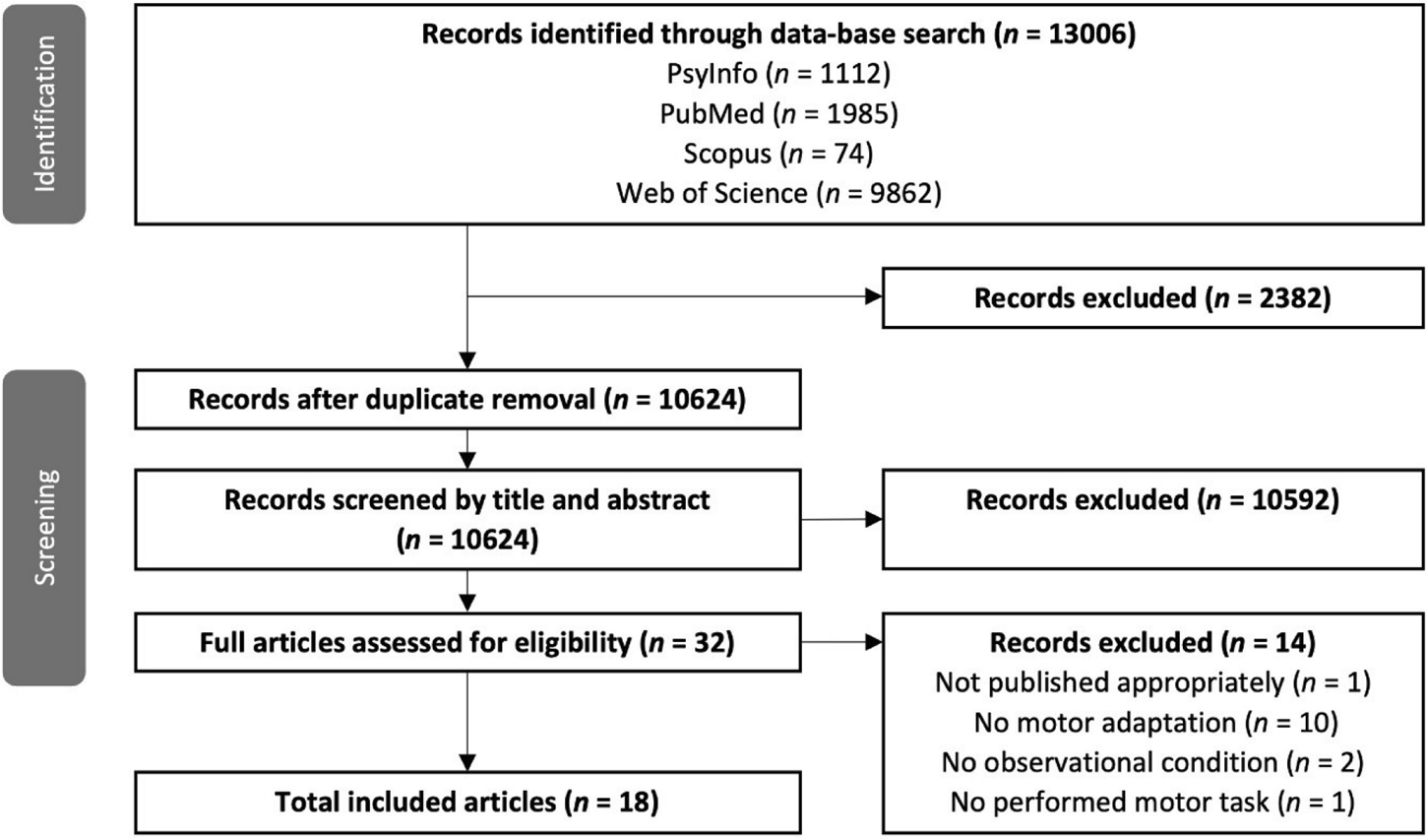
PRISMA flow chart. Number of records identified in the search process and records excluded during every step of the screening process.

In total 13,006 records were identified in all databases. After duplicate removal 10,620 studies remained for screening (c.f., Fig. 1 for a flow chart of the screening process). During full-text screening (*n* = 32), one study was excluded because it was a dissertation thesis that included studies that did not undergo peer review^25^. Further, a study from Malfait, et al. ^26^ on brain activation (fMRI) during observation of reaching errors was excluded because they only measured brain function but not behavioural performance following observational practice. Moreover, eight studies were excluded because they investigated sequence learning^27–34^; two studies^19,35^ were excluded because they investigated motor adaptation following physical, but not observational practice; one study was excluded because it investigated imitation in a neurocomputational model of the mirror neuron system^36^; and one study was excluded since it investigated interpersonal perception and dyadic coupling in a joint action scenario^37^. Our subsequent snowballing procedure did not result in the inclusion of additional studies. The final number of eligible studies was *n* = 18.

### Study characteristics

The main characteristics of the included studies can be found in Table 1. In total, 1029 young adults were included. All studies examined exclusively right-handed participants. The percentage of female participants (not reported in 4 studies and only partially reported in 1 study) was higher than 51.7% and lower than 73.1%. All studies were conducted as a randomized controlled trial or quasi experimental trial design.

**Table 1:** Characteristics of all included studies

Study ID		Participants				
Authors (year)	Study design	Device	Sample size	Female, %	Mean age [Age range]	Handedness
Bernardi et al. ^38^	RCT	Manipulandum	42	/	20.6 [SD = 2.7]	R^a^
Brown et al. ^39^	Quasi experimental trial	Manipulandum	54	/	NA	R^a^
Brown et al. ^40^	Quasi experimental trial	Manipulandum	111	/	Undergraduate students	R^a^
Larssen et al. ^49^	RCT	Graphical tablet	32	56.3	21.8 [SD = 1.6]	R^a^
Larssen et al. ^50^	RCT	Graphical tablet	93	73.1	23.0 [SD = 5.6]	R^a,b^
Lei, et al. ^54^	RCT	Exo-skeleton	40	42.5	NA [18 - 30]	R
Lim et al. ^51^	RCT	Graphical tablet	49	61.2	21.6 [SD = 3.7]	R^b^
Mattar and Gribble^41^	RCT	Manipulandum	84	52.4	21.0 [SE = 0.4]	R^a^
McGregor et al. ^42^	Quasi experimental trial	Manipulandum	30	60	22.7 [SE = 0.9]	R^a^
McGregor et al. ^43^	Quasi experimental trial	Manipulandum	112	64.3	20.7 [SE = 0.4]	R^a^
McGregor et al. ^44^	Quasi experimental trial	Manipulandum	30	60	22.7 [SE = 0.9]	R^a^
McGregor, et al. ^45^	Quasi experimental trial	Manipulandum	78	68.0	21.3 [SE = 0.6]	R^a^
McGregor, et al. ^46^	Quasi experimental trial	Manipulandum	32	71.9	21.5 [SE = 0.7]	R^a^
Ronchi et al. ^55^	Quasi experimental trial	Reaching board	66	67.0	27.4 [18 - 55]	R
Ong et al. ^52^	RCT	Graphical tablet	30	53.3	25.8 [SD = 4.1]	R^b^
Ong et al. ^53^	RCT	Graphical tablet	29	51.7	23.2 [SD = 5.6]	R^a^
Wanda et al. ^47^	Quasi experimental trial	Manipulandum	50	60	[18-38]	R^b^
Williams and Gribble^48^	RCT	Manipulandum	60	/	18.5 [SE = 1.0]	R^c^

*RCT* randomized controlled trial, *SD* standard deviation, *SE* standard error, *R* right handed.

^a^self-reported.

^b^Edinburgh Handedness Inventory^87^.

^c^Dutch Handedness Questionnaire^88^.

### Study designs

Study designs differed across studies depending on the research questions. Information about intervention groups and conditions for each study are listed in Table 2.

**Table 2:** Study groups and conditions

Authors (year)	Groups (*N*)	Observation/Practice condition (number of trials/observed movements)	Familiarisation (number of trials)	Pre-test (Number of trials)	Post-test direction of perturbation (Number of trials)
**Bernardi et al**. ^51^	Obs_Cong correct order (14)^A; Fig 5b^	Observing gradual adaptation to CCWFF (140)	No perturbation (NA)	Null field (100)	CCWFF (150)
	Obs_Incong correct order (14)^B; Fig 5b^	Observing gradual adaptation to CWFF (140)			
	Obs_Control random order (14)^C; Fig 5b^	Observing random adaption to CCWFF (140)			
**Brown et al**. ^41^	Obs_Cong correct order (12)^A; Fig 2a^	Observing gradual adaptation to CWFF (96)	/	Null field (92)	CWFF (192)
	Obs_Cong correct order/TMS-M1 (6)	Observing gradual adaptation to CWFF with M1 TMS-stimulation (96)			
	Obs_Cong correct order/TMS-SFL (6)	Observing gradual adaptation to CWFF with SFL TMS-stimulation (96)			
	Obs_Incong correct order/Control (12)^B; Fig 2a^	Observing gradual adaptation to CCWFF (96)			
	Obs_Cong correct order/rTMS-M1 (6)	Observing gradual adaptation to CCWFF with M1 TMS-stimulation (96)			
	Rest_Control (12)^C; Fig 2a^	Rest			
**Brown et al**. ^42^ ***Experiment 1***	Obs_Cong correct order (9)^A; Fig 3b^	Observing gradual adaptation to CWFF (96)	/	Null field (96)	CWFF (192)
	Obs_Cong random order (9)	Observing random adaptation to CWFF (96)			
	Obs_Incong correct order (9)^B; Fig 3b^	Observing gradual adaptation to CWFF (96)			
	Obs_Incong random order (9)	Observing random adaptation to CCWFF (96)			
	Obs_Mix random order (9)^C; Fig 3b^	Observing random adaptation to random FF (96)			
***Experiment 2***	Obs_Cong untrained (11)	Observing only high error adaptation to CWFF (96)	/	Null field (96)	CWFF (192)
	Obs_Cong random order (11)	Observing random adaption to CWFF (96)			
	Obs_Cong trained (11)	Observing only low error adaptation to CWFF (96)			
	Obs_Incong untrained (11)	High error trials learning observation (96)			
	Obs_Incong random order (11)	Observing random adaption to CCWFF (96)			
	Obs_Incong trained (11)	Observing only low error adaptation to CCWFF (96)			
**Larssen et al**. ^87^	Obs_Mix trained (8)	Observing only low error adaptation to CW (150), followed by CCW rotation (150)	No Perturbation (NA)	No Perturbation (50)	1) CW rotation (50) after second adaptation 2) CCW rotation (50) after first post-test
	Obs/Act_Mix trained (8)	Physical practice of CW rotation (150), followed by observing only low error adaptation to CCW rotation (150)			
	Act_Cong no CCW (8)	Physical practice of CW rotation (150)			
	Obs/Act_Mix (8)	Physical practice of CW rotation (150), followed by physical practice of CCW rotation (150)			
**Larssen et al**. ^88^	Obs/Act trained/pre obs (19)^A4^	Observing only low error adaptation to CCW rotation (50), followed by physical practice of CW rotation (50)	No perturbation (20)	No perturbation (20)	1. No perturbation (20), after first adaptation phase 2. No perturbation (20), after second adaptation phase 3. CW perturbation (20), after second post-test 4. No perturbation (20), after 24 hours 5. CW perturbation (20), after third post-test
	Obs/Act trained/post obs (18)	Physical practice of CW rotation (50), followed by observing only low error adaptation to CW rotation (50)			
	Obs/Act trained/interspersed (18)	Interspersed observing only low error adaptation to CW rotation and physical practice of CW rotation (2x50, switch every five trials)			
	Act_Cong (19)^C; D Fig2/Adaptation 1/Block1; Fig5/ Post-Test 1/Block 1^	Physical practice of CW rotation (2x50)			
	Rest_Cong (18)	Interspersed rest and physical practice of CW rotation (2x25, rest after every five trials)			
**Lei et al**. ^58^	Act_Cong (8)	Physical practice of CW rotation (120)	/	No perturbation (60)	CCW rotation (80)
	Obs_Cong (8)	Observing gradual adaptation to CW rotation (120)			
	Pro (8)	Passive reaching in CCW rotation with active return to starting point (120)			
	Obs/Pro interspersed (8)	Interspersed observing adaptation to CCW rotation and passive reaching in CCW rotation with active return to starting point (120, switch every 20 trials)			
	Rest_Control (8) ^C; Fig 3a^	Rest			
	Pro2 (7)	Passive reaching in CCW rotation with passive return to starting point (120)			
**Lim et al**. ^55^	Obs/Act trained/CW (14)^D; Fig 2/Phase 2/Post-test 2/B1^	Physical practice of CW rotation (150), followed by observing only low error trial adaptation to CW rotation (150)	No perturbation (25)	No perturbation (50)	1. No perturbation (50), after first adaptation 2. No perturbation (50), after second adaptation 3. CW rotation (50), after second post-test
	Obs/Act trained/CCW (11)^E; Fig 2/Phase 2/Post-test 1/B1^	Physical practice of CCW rotation (150), followed by observing only low error trial adaptation to CCW rotation (150)			
	Act_Control (12)	Physical practice of CW rotation (150)			
	Obs_Cong trained (12)^A; Fig 2/Phase 2/Post-test 2/B1^	Observing adaptation to CW rotation (150)			
**Mattar and Gribble**^45^ ***Experiment 1***	Obs_Cong correct order (12)^A; Fig 4a^	Observing gradual adaptation to CWFF (96)	No perturbation (96)	/	CWFF (192)
	Obs_Incong correct order (12)^B; Fig 4a^	Observing gradual adaptation to CCWFF (96)			
	Rest_Control (12)^C; Fig 4a^	Rest			
***Experiment 2***	Obs_Mix random order (12)	Observing random adaptation to random FF (96)	No perturbation (96)	/	CWFF (192)
***Experiment 3***	Obs_Cong correct order/distraction task (12)	Observing gradual adaptation to CWFF with arithmetic distraction task (96)	No perturbation (96)	/	CWFF (192)
***Experiment 4***	Obs_Cong correct order/movement task (12)	Observing gradual adaptation to CWFF with simple arm movement task (96)	No perturbation (96)	/	CWFF (192)
	Act_Control (12)	Simple arm movement task (move arm in circular motion) for 12 min (length of the learning video)			
**McGregor et al**. ^53^ ***Experiment 1***	Obs_Incong (15)^B; Fig 4b^	Observing gradual adaptation to CCWFF (200)	No perturbation (50)	No perturbation (200)	CWFF (100)
***Experiment 2***	Obs_Mix random order (15)^C^	Observing random adaptation to random FF (200)			
**McGregor et al**. ^46^ ***Experiment 1***	Obs_Incong gradual order (16)^B; Fig 1c^	Observing gradual adaptation to CCWFF (200)	/	No perturbation (50)	CWFF (50)
	Obs_Incong gradual order/ stimulation/right (16)	Observing gradual adaptation to CCWFF with median nerve stimulation of the right arm (200)			
	Obs_Incong gradual order/ stimulation/left (16)	Observing gradual adaptation to CCWFF with median nerve stimulation of the left arm (200)			
	Obs_Incong gradual order/ stimulation/both arms (16)	Observing gradual adaptation to CCWFF with median nerve stimulation of both arms (200)			
	Obs_Mix random order (16)^C; Fig 1c^	Observing random adaptation to random FF (200)			
***Experiment 2***	Obs_Incong gradual order/stimulation /right/S1 SEP (16)	Observing gradual adaptation to CCWFF with median nerve stimulation of the right arm (200), somatosensory evoked potential assessment by EEG of S1 before and after observation		No perturbation (50)	CWFF (50)
	Obs_Mix random order/S1 SEP (16)	Observing random adaptation to random FF (200), somatosensory evoked potential assessment by EEG of S1 before and after observation			
**McGregor et al**. ^52^	Obs_Incong gradual order (15)^B; Fig 2b^	Observing gradual adaptation to CCWFF (200)	No perturbation (50)	No perturbation (200) (BL)	CWFF (100)
	Obs_Mix random order (15)^C; Fig 2b^	Observing random adaptation to random FF (200)			
**McGregor et al**. ^43^	Obs_Cong gradual order/perception feedback (26)^A; Fig 3b^	Observing gradual adaptation to CCWFF (200), with perception feedback	No perturbation (30)	No perturbation (50)	CCWFF (50)
	Obs_Cong gradual order/no perception feedback (26)	Observing gradual adaptation to CCWFF (200), without perception feedback			
	Obs_Mix random order/perception feedback (26)^C; Fig 3b^	Observing random adaptation to random FF (200), with perception feedback			
McGregor, et al. ^46^	Obs_Incong gradual order (16)^B; Fig 3c^	Observing gradual adaptation to CWFF (192)	No perturbation ( ~ 40)	No perturbation (96)	CCWFF (96)
	Obs_Mix random order (16)^C; Fig 3c^	Observing random adaptation to random FF (192)			
Ong et al. ^52^	Act_Cong No Hand (10)	Physical adaptation to CW rotation without hand vision	No perturbation (10-20)	No perturbation (50)	1. No perturbation (50) 2. CW rotation (50) (after post-test, Obs Live only)
	Act_Cong Hand (10)^C; Fig 4/Block C1; D; Fig 3^	Physical practice of adaptation to CW rotation with hand vision (200)			
	Obs_Cong Live (10)^A; Fig 4/Block C1^	Observing gradual adaptation to Act_CLG NoHand with hand vision live (200)			
Ong et al. ^53^	Obs trained/Hand/No Hand (10)^A; Fig 1/Post 2a^	Observing only low error adaptation to CW rotation with hand vision (150), and without hand vision (50)	No perturbation (NA)	No perturbation (50)	1. No perturbation (50) (after adaptation) 2. CW rotation (50) (after first post-test) 3. No perturbation (50) (after second post-test)
	Obs/Act trained/Hand/No Hand (9)	Physical practice of adaptation to CW rotation without hand vision (50), followed by observing only low error adaptation to CW rotation with hand vision, and without hand vision (25)			
	Act_Control Hand/No Hand(10)^C; Fig1/B1; DFig 1/Post 1a^	Physical practice of adaptation to CW rotation with hand vision (150), and without hand vision (50)			
**Ronchi et al**. ^59^ ***Experiment 1***	Obs_const Live (12)	Observing reaches with constant error (60)	/	No perturbation (10)	No perturbation (10)
***Experiment 2***	Obs_const Live (22)	Observing reaches without error (20), followed by observing reaches with constant error (60)	/	No perturbation (10)	No perturbation (10)
	Obs gradual order (22)	Observing reaches without error (20), followed by observing gradual adaptation to rightward error (60)	/		
Wanda et al. ^47^ ***Experiment 1***	Act_vis (10)	Physical practice of gradual adaptation to viscous CWFF (192)	/	No perturbation (96) Interspersed force –channel (16)	Force-channel (24)
	Act_stiff (10)	Physical practice of gradual adaptation to stiff CWFF (192)			
	Obs_vis (10)	Observing gradual adaptation to viscous CWFF (192)			
	Obs_stiff (10)	Observing gradual adaptation to stiff CWFF (192)			
***Experiment 2***	Obs_stiff (10)	Observation of gradual adaptation to stiff CWFF (2x96, 3 min rest between)	/	No perturbation (96) Interspersed force –channel (16)	Force-channel (24)
Williams and Gribble^48^	Obs_Cong right (15)^A; Fig 3^	Observing adaptation learning to CWFF with right hand (96)	/	No perturbation (96)	CWFF with right hand (96)
	Obs_Cong left (15)	Observing adaptation learning to CWFF with left hand (96)			
	Obs_Incong right (15)^B; Fig 3^	Observing adaptation learning to CCWFF with right hand (96)			
	Obs_Incong (15)	Observing adaptation learning to CCWFF with left hand (96)			

The first abbreviation of the group indices describe the adaptation period condition with a pure *Obs* Observational practice group, *Act* Physical practice group, *Rest* rest during adaptation period, *Pro* Proprioceptive practice group; The second abbreviations of the group indices describe the practice condition and therefore, the characteristics of the perturbation in reference to the post-test condition with *Cong* congruent stimulus, practice group with congruent adaptation stimulus, *Incong* incongruent stimulus, practice group with incongruent adaptation stimulus, *MS* mixed stimulus, learning group with multiple practice stimuli, *Control* control group, *vis* velocity dependent force field, *stiff* position dependent force field; the order could be either gradual or random and reflects how the visual stimuli were presented if the video showed a participant who gradually declined the reaching error or a random process of high and low reaching errors and force-field directions; abbreviations for practice conditions denote *CW* clockwise or *CCW* counterclockwise and *FF* Force-Fields; Letters in superscript refer to the group assignment in Table 5 and Table 6 and the figures the data was extracted from: A = Obs_Cong, B = Obs_Incong, C = CG, D = Act_Cong, E = Act_Incong.

Reaching was operationalized by three different types of devices (see Table 1): (i) *n* = 11 studies used robotic manipulanda where participants had to move a lever to different positions through a force-field^38–48^. All 11 studies applied a velocity dependent force field where higher reaching velocities resulted in larger external resistance. Wanda, et al. ^47^ additionally applied a position dependent force-field where the resistance of the force-field was bound to the hand position on the manipulandum plane; (ii) *n* = 5 studies used graphic tablets where participants moved a pointing device (attached to their index fingers) to different positions on a graphic tablet^49–53^ while the visual feedback of hand-traces was rotated by 30° in clockwise or counterclockwise direction; (iii) *n* = 1 study^54^ used a robotic exoskeleton with the reaching arm supported by a kinetic arm, where they had to perform reaches to targets displayed on a screen that was placed atop of the reaching arm with 30° perturbations; (iv) *n* = 1 study^55^ used an LED mounted on a pulley on the far side of a test box to track the participants’ hand positions. A metal thimble attached to the right index finger served as a reference point. The participants were required to reach towards a downward projected position of the red LED, which was positioned in front of their body midline.

Sixteen studies asked participants to perform reaches with the according testing device to targets, projected on a semi-silvered mirror that was mounted atop of the apparatus and one study used a regular screen in a similar position. Hand position was represented by a cursor or a circular marker on the semi-silvered mirror or screen. Ronchi, et al. ^55^ used a black test box opened on the side, facing the participant. The LED was attached to a pulley positioned on the distal side of the test box. Reaching tasks were performed exclusively with the right hand across all studies. The number of reaches differed between 10 and 192. Further, in *n* = 17 studies participants were asked to complete the task in a pre-defined time-window and received feedback whether this was met. These time-windows ranged from 150 ms to 1500 ms. In 12 studies, movement duration feedback was provided by a colour change of the target^38,41–49,51,53^, two studies reported they gave verbal feedback^50,52^, and another three studies did not specify their feedback^39,40,55^. In case trials did not meet the velocity threshold, they were excluded in five studies^49–51,53,54^, whereas all other studies did not exclude trials based on this criterion.

Eleven studies^38,42–47,49–51,54^ marked start and target position with coloured circles, one study^55^ marked the target position with a red LED, and six studies^39–41,48,52,53^ did not report how target positions were marked.

With respect to instructions, in six studies participants were asked to reach to the target in a line as straight as possible^38,42–46^; in another eight studies they were instructed to make “fast and accurate” movements to the target^39–41,47,48,51,53,54^; in three studies they were asked to consider both, the velocity and accuracy constraint at the same time^49,50,52^; in the study by Ronchi, et al. ^55^, no standardised instructions were reported.

Observational practice was conducted across studies by use of video material across all studies except for Ronchi, et al. ^55^, where the subjects observed live reaching movements performed by the experimenter. However, discrepancies between studies were found with respect to various aspects of the stimuli presented for the purpose of observational practice.

In the study by Ronchi, et al. ^55^, participants observed live reaches of the experimenter reaching to different dots on a reaching board either with a constant rightward error or a gradual performance change (gradual reduction of an initially large error) over time. In two studies^52,53^ subjects in the observer group were paired with subjects in the physical practice group so that each observer viewed an entire practice session of their partner; all other studies showed recorded videos.

All studies presented video material that showed the arm of an actor from a top-down perspective except of one that did not report this^49^. The sequence of videos that was presented to the observer either displayed gradual performance changes over time (i.e., a learning curve)^38–40,42–48,52,54^, only trials with high errors (early learning phase)^40^, trials with low errors (late learning phase)^50,51,53^, or trials with a constant error Ronchi, et al. ^55^. Eight studies showed a mix of trials with actors compensating both clockwise and counterclockwise perturbations as an unlearnable control condition^40–46^. Observed practice trials ranged between 60 and 200 reaches (cf. Table 2). The studies’ respective video material was repeated twice across four studies^39,41,46,47^, five times in Bernardi, et al. ^38^, and only once for the rest of the studies.

To ensure attentional focus during observational practice, five studies asked participants to report the sum of correct timing trials after every block^42–46^. In five studies, participants were asked to report if the actor trials were too fast or too slow^38–41,48^. In one study the participants were asked to pay close attention^52^ and in another one to engage in imagery^53^. Five studies did not report any measures to assure that participants paid attention to the videos^47,49–51,54^. Observers received direct feedback on the actor’s movement times indicated by a colour change for either too fast or too slow movements. All studies used a first-person perspective, so it seemed like they would observe their own reaches. Solely, Wanda, et al. ^47^ reported that participants were required to hold the handle of the robotic manipulandum during the observation phase.

All studies incorporated at least one group that learned purely through action observation. Depending on their research question, however, they contrasted the results of the action observation group to a variety of different other groups or conditions that either observed a modulated sequence of videos (observational control groups) or performed physical practice (physical practice control groups).

Control groups either did not receive any stimulus presentation, but rather rested for the same amount of time as the observational practice group^39,41,54^; they investigated random force-fields where actors practiced both, clockwise and counterclockwise force-fields in an interspersed manner^40,42–44^; or they observed a scrambled sequence of practice trials that displayed all practice trials of an actor in a random sequence rather than from high to low error trials^38,40^. One study^41^ investigated a group that received a secondary distracting task during action observation where participants had to sum up a number of digits that were displayed during observational practice.

Physical practice groups either performed pure physical practice^49,50,52–54^ or interspersed physical with observational practice. One study introduced a group where participants were provided with observational practice before physical practice^50^, whereas three studies had groups that observationally practiced the task after physical practice^49,51,53^. Additionally, one study had two interspersed groups, where the participants practiced five trials physically, combined with five trials by observation or just rest during the observation trials^50^. Moreover, Ong and Hodges^52^ differentiated groups by occluding vision of the hand while practicing in the perturbed environment in one out of two physical practice groups. Participants with occluded vision of the hand could only see the cursor feedback on the screen while adapting their reaches to the visuomotor perturbation. In their second study^53^, they used mixed approaches, performing some trials with visual feedback of the hand and some without.

Typically, participants observed an actor performing the reaching task in a perturbed condition that was congruent to the testing condition (i.e., the same visuomotor rotations or force-field perturbations). In nine studies^39–44,46,48,51^, however, participants observed an actor performing in a condition (e.g., clockwise perturbation) that was incongruent to the testing condition (e.g., counterclockwise perturbation).

All studies, except for Ronchi, et al. ^55^ tested for direct effects of observational practice in a perturbed environment. Six studies^49–53,55^ tested for aftereffects (reaching errors resulting from different practice modalities measured in a post-test) in a non-perturbed environment as a measure of changes to internal models following observational practice. Three of the five studies introduced a second post-test, where the participants had to perform reaches in a perturbed environment, after being tested for aftereffects^50,52,53^.

Outcome measures to assess learning or adaptation following observational practice focused on how movement trajectories deviated from a straight line. Nine out of 11 studies that used the robotic manipulandum^39–46,48^ extracted an outcome of movement curvature which is defined as the maximum perpendicular deviation from a straight line (termed either trajectory curvature, movement curvature or maximum perpendicular deviation). In the study by Bernardi, et al. ^38^ movement curvature was extracted at peak speed. And in the study by Wanda, et al. ^47^, the integrated force (Ns) was recorded with which participants pushed the manipulandum in lateral direction to compensate for the perturbation (in the testing environment it was only possible to move the manipulandum in a straight line from the start to the target). All studies that used visuomotor rotation paradigms^49–54^ computed the directional error which is defined as the directional distance (in degrees) between actual trajectories and desired trajectories at the timepoint of maximum velocity. One study^55^ investigated the proprioceptive shift of their body midline, visual shift and visual-proprioceptive shift after observational learning. The shift was computed by the subtraction of post from pre-measures of all three variables.

A total of five studies used additional methods of neuroimaging or non-invasive brain stimulation to identify neural correlates and investigate brain mechanisms of observational learning. McGregor and Gribble^42,44^ used functional magnetic resonance imaging (fMRI) to investigate resting state connectivity between visual sensory and motor areas of the brain as correlates of learning. McGregor, et al. ^46^ used transcranial magnetic stimulation (TMS) to probe whether observational practice changes corticomotor projections from the primary motor cortex (M1) to the right hand. Brown, et al. ^39^ used repetitive TMS (rTMS) over M1 following observational practice to investigate how inhibition of this area affects learning. McGregor, et al. ^43^ performed peripheral nerve stimulation of the median nerve to investigate whether observational practice changes somatosensory excitability.

### Risk of bias

The RoB 2.0 tool has been used to assess the risk of bias for all included studies. A summary of all risk of bias assessment for all domains and an overall risk of bias judgement can be found in Table 3. Overall, the studies had either a high risk of bias (*n* = 8) or at least some concerns of bias (*n* = 9).

**Table 3:** Risk of bias judgement for each included study based on the Cochrane RoB2 tool

Authors (Year)	Bias arising from the randomisation process	Bias due to deviations from the intended intervention	Bias due to missing outcome data	Bias in measurement of the outcome	Bias in selection of the reported result	Overall risk of bias judgement
Bernardi et al. ^38^	Some concerns	Low risk	Low risk	Low risk	Some concerns	Some concerns
Brown et al. ^39^	High risk	Some concerns	Low risk	Some concerns	Some concerns	High risk
Brown et al. ^40^	Some concerns	Some concerns	Low risk	Low risk	Some concerns	High risk
Larssen et al. ^49^	Some concerns	Low risk	Low risk	Low risk	Some concerns	Some concerns
Larssen et al. ^50^	Some concerns	Low risk	Low risk	Low risk	High risk	High risk
Lei et al. ^58^	Some concerns	Low risk	Low risk	Low risk	Some concerns	Some concerns
Lim et al. ^51^	Some concerns	Low risk	Low risk	Low risk	Some concerns	Some concerns
Mattar and Gribble^45^	Some concerns	Low risk	Low risk	Low risk	Some concerns	Some concerns
McGregor and Gribble^53^	High risk	Low risk	Low risk	Low risk	Some concerns	High risk
McGregor et al. ^46^	High risk	Some concerns	High risk	Low risk	Some concerns	High risk
McGregor and Gribble^52^	High risk	Low risk	Low risk	Low risk	Some concerns	High risk
McGregor et al. ^43^	High risk	Low risk	Low risk	Low risk	Some concerns	High risk
McGregor et al. ^47^	Some concerns	Low risk	Low risk	Low risk	Some concerns	Some concerns
Ong and Hodges^57^	Some concerns	Low risk	Low risk	Low risk	Some concerns	Some concerns
Ong et al. ^56^	Some concerns	Low risk	Low risk	Low risk	Some concerns	Some concerns
Ronchi et al. ^59^	Some concerns	Low Risk	Low Risk	Low risk	Some concerns	Some concerns
Wanda et al. ^48^	High risk	Low risk	Some concerns	Low risk	Some concerns	High risk
Williams and Gribble^44^	Some concerns	Low risk	Low risk	Low risk	Some concerns	Some concerns

### Results of individual studies

**Fig. 2:**
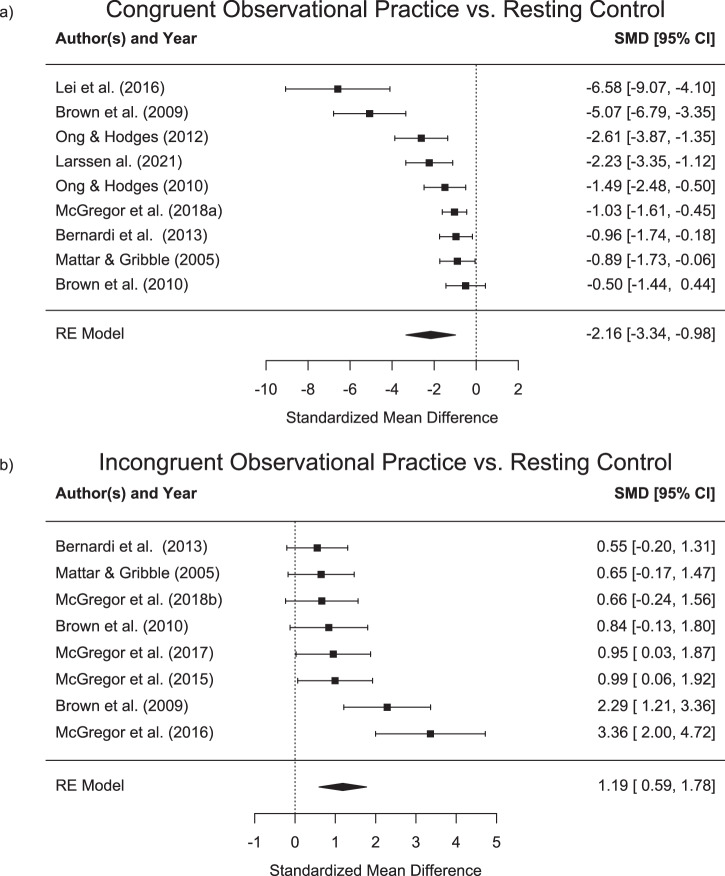
Direct effects of observational practice vs. resting control. Standardised mean differences (Cohen’s d) and confidence intervals (CI) for direct effects of observational learning against a naive control group or observational practice in an unlearnable environment. Upper panel (**a**) displays results in congruent observational practice with negative values indicating an error decrease. Lower panel (**b**) displays results in incongruent observational practice with positive values indicating an error increase.

Nine studies showed decreased movement curvature or directional error for participants when moving in a perturbed environment following congruent observational practice as compared to a resting control group or a group that practiced in an unlearnable environment^38–41,45,50,52–54^. In addition, eight studies found an increased curvature or directional error following observational practice of an incongruent force field or visuomotor rotation^38–44,46^. Standardized effect sizes for the different groups are shown in Table 4. Forest plots with standardized mean differences and random effects model results between congruent (upper panel) and incongruent (lower panel) observational practice and the respective control groups are shown in Fig. 2.

**Table 4:** Direct effects of observational practice in comparison to control groups

Authors	Control *M (SD)*	Obs_Cong *M (SD)*	Obs_Incong*M (SD)*
Bernardi et al. ^51^	3.5 (0.6)	2.8 (0.8)	4.3 (1.9)
Brown et al. ^41^	13.1 (1.6)	4.9 (1.5)	16.6 (1.3)
Brown et al. ^42^	15.9 (3.8)	14.1 (3.0)	18.8 (2.7)
Larssen et al. ^88^	22.9 (1.1)°	19.7 (1.6)°	/
Lei, et al. ^58^	26.7 (0.9)°	19.7 (1.1)°	/
Mattar and Gribble^45^	15.0 (3.4)	11.2 (4.7)	17.6 (4.3)
McGregor et al. ^53^	31.5 (8.8)	/	41.0 (9.5)
McGregor et al. ^46^	3.7 (7.9)	/	36.9 (10.8)
McGregor et al. ^52^	32.2 (8.3)	/	41.1 (9.6)
McGregor, et al. ^43^	31.5 (9.1)	23.4 (6.07)	/
McGregor, et al. ^47^	9.4 (2.6)	/	11.5 (3.4)
Ong et al. ^57^	22.1 (9.4)°	6.1 (9.1)°	
Ong et al. ^56^	19.6 (12.0)°	−1.2 (6.3)°	
Wanda et al. ^48^	/	19.9 (7.0)	25.2 (4.4)

Error scores (mean and SD) are detailed for the different groups. Smaller errors for the congruent, and larger errors for the incongruent learning group as compared to the control group indicate direct effects from observational practice.

*M* Mean, *SD* Standard deviation, *Control* Control group (rest or observational practice of an unlearnable environment), *Obs_Cong* Observational practice group with congruent adaptation stimulus, *Obs_Incong* Observational practice group with incongruent adaptation stimulus; All means and standard deviations are reported in millimetres (mm) or degree (°).

**Fig. 3:**
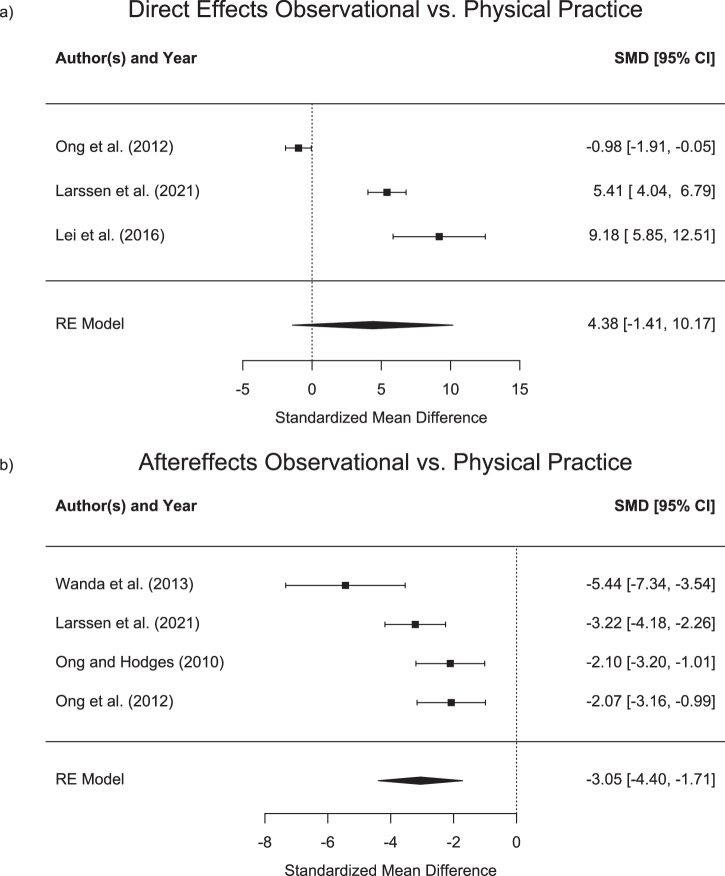
Direct- and aftereffects of observational vs. physical practice. Standardised mean differences (Cohen’s d) and confidence intervals (CI) for direct effects (**a**) and aftereffects (**b**) of observational vs. physical practice. For direct effects, positive values indicate larger effects for physical practice. For aftereffects, larger negative values indicate higher effects for physical practice.

In addition, three studies allowed for the comparison of the direct effects following observational and physical practice^50,53,54^. While Ong, et al. ^53^ found similar direct effects for both, physical and observational practice, both Larssen, et al. ^50^ and Lei, et al. ^54^ found significantly larger effects of physical practice. Mean and standard deviations for the direct effects in the different learning groups are shown in Table 5 and forest plots with standardized mean differences are displayed in Fig. 3 (upper panel).

**Table 5:** Direct effects (mean and SD) for the congruent observational learning and physical practice groups

Authors	Obs_Cong *M (SD)*	Act_Cong*M (SD)*
Ong et al. ^56^	−2.35 (3.50)°	1.39 (3.80)°
Larssen et al. ^88^	19.75 (1.58)°	12.66 (0.89)°
Lei et al. ^58^	19.75 (1.11)°	6.54 (1.57)°

*M* Mean, *SD* Standard deviation, *d* Obs_Cong, Observational practice with congruent adaptation stimulus, *Act_Cong* Physical practice group with congruent adaptation stimulus; all means and standard deviations are reported degrees (°).

Three studies demonstrated unlearnable environments (unsystematic changes in the observed task-error) which displayed actors practicing in both clockwise and counterclockwise force-fields in an interspersed manner^42–44^. In addition, two studies investigated whether the display of a randomly scrambled sequence of all practice trials of one actor led to different performance improvements than a fixed sequence. While Bernardi, et al. ^38^ found that a scrambled sequence led to reduced learning, Brown, et al. ^40^ did not find any difference between scrambled and fixed sequences. While participants in the study by Brown, et al. ^40^ observed and learned the novel task constraints (clockwise or counterclockwise perturbation) in eight different directions, those in Bernardi, et al. ^38^ only observed and were tested in one direction. Consequently, participants in Brown, et al. ^40^ observed a larger proportion of high-error trials as actors adapted slower to the perturbation. Moreover, Brown, et al. ^40^ additionally showed that observing high-error trials is more beneficial for learning than watching solely low-error trials. Investigating effector dependence of observational learning, Williams and Gribble^48^ found that learning effects are independent of whether actors are observed that perform the task with the right- or left-hand when tested with the right hand.

Four studies^49–51,53^ have investigated the effect of mixed schedules on observational practice. While Ong, et al. ^53^ have shown no differences between two groups that received either physical or observational practice as well as a third group, receiving a mixed schedule, all other three studies have indeed shown differential effects of mixed schedules. Larssen, et al. ^49^ showed that participants in the mixed group show less error when tested following practice in a clockwise perturbation and then, in a subsequent test in a counterclockwise perturbation. Similarly, Lim, et al. ^51^ have demonstrated that a mixed practice group showed larger direct effects as compared to pure physical practice group in a subsequent retention test in a perturbed environment. Finally, Larssen, et al. ^50^ have demonstrated largest direct learning effects in a mixed group that observed and physically practiced in an interspersed manner, followed by a group that performed pure practice and smallest direct effects for a pure observational practice group. In summary, studies showed that observational practice leads to motor adaptation that goes beyond the effects of a resting control group. The effect sizes for direct learning effects from observational practice from congruent stimuli ranged from d = −0.5 to d = −5.07, with an overall effect size of d = −1.71, indicating an error reduction as compared to a control group. As opposed to that, effect sizes for incongruent stimuli ranged from d = 0.55 to d = 3.36 with an overall effect size of 1.19, showing an error increase. However, in comparison to physical practice, two out of three studies found larger adaptation for physical practice with effect sizes between 5.41 and 9.81. One study found no difference with an effect size of 0.98. The overall effect size was 4.38 in favour of physical practice.

Six studies assessed aftereffects resulting from observational practice^47,50–53,55^. All studies except for Ronchi, et al. ^55^ compared aftereffects following observational to those of physical practice. Group means and standard deviations for aftereffects following observational and physical practice are shown in Table 6. All studies demonstrated strong aftereffects (i.e., deviations from a straight line in a non-rotated environment) following physical but small or absent aftereffects following observational practice. Standardized mean differences in aftereffects between physical and observational practice are displayed in the forest plots in the lower panel of Fig. 3. Due to the missing physical practice group in Ronchi, et al. ^55^ and due to the lack of a dispersion metric in Lim, et al. ^51^, standardized mean differences could only be calculated for the remaining 4 studies.

**Table 6:** Aftereffects (mean and SD) for the congruent observational learning and physical practice groups

Authors	Obs_Cong *M (SD)*	Act_Cong *M (SD)*
Larssen et al. ^88^	3.2 (5.0)°	17.8 (3.8)°
Lim et al. ^55^	1.00°	20.3°
Ong and Hodges^57^	2.1 (1.6)°	7.94 (3.4)°
Ong et al. ^56^	2.1 (1.6)°	11.17 (5.7)°
Wanda et al. ^48^	0.1 (0.1)Ns	1.37 (0.3)Ns

*M* Mean, *SD* Standard deviation, *d* Obs_Cong, Observational learning with congruent adaptation stimulus, *Act_Cong* Physical practice group with congruent adaptation stimulus; all means and standard deviations are reported degrees (°) or Impulse (Ns).

Studies identified different modalities that affected the magnitude of aftereffects following physical or observational practice. Ong and Hodges^52^ have shown that an occluded vision of the hand during physical practice leads to attenuated aftereffects as compared to physical practice with vision of the own hand. Regardless of whether the hand was visible or not, however, aftereffects following physical practice were always greater as compared to observational practice. When investigating mixed schedules, Ong, et al. ^53^ showed larger aftereffects for a group that received a mix of observational and physical practice, as compared to pure physical or observational practice. In contrast, Larssen, et al. ^50^ found the opposite result with a mixed group showing reduced aftereffects as compared to a pure physical practice group.

Due to the absence of a control group that received physical practice, Ronchi, et al. ^55^ was not included in Fig. 3. In the study, it was found that observing a rightward constant reaching error results in leftward visual and proprioceptive alterations of their sensed body midline (which can be considered aftereffects). In summary, studies showed larger aftereffects for physical as compared to observational practice, with effect sizes ranging between d = −2.07 to d = −5.44 and an overall effect size of d = −3.05.

Brown, et al. ^39^ have used non-invasive brain stimulation (rTMS) to inhibit M1 during observational practice. They revealed a reduced trajectory curvature following incongruent, and an increased trajectory curvature following congruent observational practice, indicating reduced learning in groups where M1 was inhibited during action observation. This suggests an involvement of this region in motor adaptation. In contrast, a control group in the same study that received rTMS to the left superior frontal gyrus (an area that is not associated with force-adaptation), did not show any differences in learning than groups without stimulation^39^. Additionally, using TMS to probe corticomotor excitability of the left M1, McGregor, et al. ^46^ found an increase in the excitability of the right hand (FDI and APB muscles) following observation of an adaptation sequence (learning condition), whereas they found no changes in the excitability following observation of a control condition where actors performed reaches in an unlearnable environment (mixed clockwise and counterclockwise trials), providing additional evidence for an involvement of M1 in observational motor adaptation.

In order to unravel the involvement of S1 in observational learning, McGregor, et al. ^43^ have demonstrated that stimulation of the median nerve during observational practice disrupted learning. In a second experiment^43^ they showed that observational practice leads to increased amplitudes for the sensory evoked potential, an effect that was not visible for a control group. They furthermore found a positive correlation with the magnitude of the amplitude change and the learning gains.

Using fMRI, McGregor and Gribble^42^ found that observational learning is correlated with changes in functional resting state connectivity between the middle temporal visual area (V5/MT) and other areas involved in sensorimotor processing, including M1, S1 and the cerebellum. Their results suggest that an increase in the connectivity between V5/MT and M1 as well as S1, as well as a decrease in the connectivity between V5/MT and the cerebellum is associated with higher learning gains. In a subsequent study, McGregor and Gribble^44^ investigated whether pre-learning functional connectivity can predict motor learning. They found that functional connectivity of a sensorimotor network linking the bilateral M1, dorsal premotor cortex (PMd), S1 and the left superior parietal lobule (SPL) was positively correlated with subsequent learning gains. They did not find any relationship between learning gains and functional connectivity prior to observational practice in the control group.

### Reporting biases and certainty of evidence

Overall, observational practice was shown to be beneficial for adaptation to perturbed reaching. The evidence was downgraded twice due to risk of bias and publication bias. Risk of bias was present with some concerns in (*n* = 10) high-risk in (*n* = 8) studies and no low-risk studies (see Table 3). Moreover, the publication bias may be present as the published studies are dominated by two main research groups who each applied one of the two main reaching paradigms. Fourteen out of the 18 studies are attributed to one of these two groups. Due to relatively big effect sizes the evidence was upgraded. Moreover, all studies had homogenous sample populations of relatively young adults. Therefore, the certainty of evidence could be stated as moderate for the populations of relatively young adults with the specific tasks. Certainty of evidence was relatively low for the neural mechanisms of observational learning as only five studies^39,42–44,46^ investigated those with different methodologies and research questions and relatively small sample sizes.

## Discussion

The aim of our study was to systematically review the literature on observational learning in reaching and aiming, to (i) compare effects between observational and physical practice; (ii) identify influencing factors of learning gains; and (iii) synthesize knowledge about the neurophysiological underpinnings of observational learning. Our systematic review has demonstrated that learners can adapt reaching/aiming skills to novel task constraints such as visuomotor rotations and force fields through observation of an actor and in the absence of physical practice. More specifically, we have concluded that (i) while observational practice leads to performance improvements (i.e., direct effects), they are typically smaller as compared to physical practice and aftereffects are smaller, suggesting different learning mechanisms as will be further elaborated in the discussion. (ii) The most important factor for learning through observation seems to be the visualisation of the task-error. (iii) With respect to neurophysiological correlates, existing studies indicate a strong involvement of the primary motor cortex and the somatosensory cortex in observational learning, thus indicating similar neural mechanisms as compared to learning from physical practice.

Our primary objective was to compare the effects of observational and physical practice. However, there were only three studies that compared the performance improvements between the two practice types (direct effects). Two studies show better performance improvements following physical practice^50,54^. Contrarily, Ong, et al. ^53^ did not find any differences between observational and physical practice.

However, aftereffects were lower after observational than physical practice. Aftereffects describe the phenomenon that after successful adaptation following physical practice, additional practice is required to re-adapt to the original unperturbed environment^19,56^. They are interpreted as a marker of implicit learning^24^ the adaptation of internal models^19^, and cerebellar involvement^21,57^. The lack of aftereffects following observational practice indicates different underlying learning mechanisms compared to physical practice. Unfortunately, it is unclear whether observational practice also leads to some, albeit small aftereffects, since there is no study that compares aftereffects between observational practice and inactive control groups.

From a motor control point of view, performance improvements following observational practice can be also discussed within the framework of internal models^20,24^. The concept of internal models is linked to the inverse problem, which is a major challenge in motor control. The problem signifies the difficulty that a certain motor output (e.g., a trajectory) must be achieved through some input (e.g., muscle forces). The question how this set of inputs is computed, given a desired output is complex and non-trivial^58^. The *inverse* internal model is the process that computes a required input pattern to achieve a specific task-goal given a set of constraints. The *forward* model, on the other hand, predicts the sensory consequences/states of an intended action and can thus be used to compare the actual with the desired movement outcome on a sensory level, therefore allowing rapid error correction in feedback control^20,21^.

Implicit and explicit learning are processes that are closely linked to the notion of internal models^24^. Previous research has demonstrated that motor adaptation can occur through implicit learning as a consequence of practice as well as explicit learning as a consequence of adopting a strategy^56^. Explicit learning has shown to speed up or result in immediate adaptation processes without the need for repeated physical practice, but without aftereffects^56,59^. The downside of explicit learning processes is the reduced temporal stability of explicit as compared to implicit processes^24,60^.

Studies included in this review have^49–53^ demonstrated that individuals who observed others had better explicit knowledge of the novel task-constraints (i.e., the direction and magnitude of the perturbation) as compared to those who physically practiced. In addition, evidence in this review has shown physical as opposed to observational practice leads to larger aftereffects. Taken together, these results suggest a higher degree of implicit learning for physical practice. Further research should identify whether observational practice also leads to unintended aftereffects, which would indicate that inverse models are also formed following observational practice.

Taken together, both observational as well as physical practice demonstrate effective performance changes when learning novel task constraints in a reaching task. However, physical practice produces more stable and sustainable performance changes and stronger aftereffects. This is potentially due to observational practice being dominated by explicit while physical practice involves both, implicit and explicit learning processes. In line with this reasoning, we suggest that observational practice rather leads to an update of inverse models while physical practice updates both, the inverse and forward model.

As an alternative explanation, it was however also brought forward that the lack of aftereffects is not per se an indication that no forward model was developed, but rather that effector independent aspects of an internal model (both, inverse and forward) were learned^61^. In line with this argument, Williams and Gribble^48^ found evidence for effector independent learning through observational practice (i.e., there is no difference in learning when observing a model reaching with the left or right hand).

In certain situations, this lack of aftereffects and more explicit knowledge about the task may be beneficial, however, for example when learning multiple (conflicting) task constraints at the same time^49^. Previous studies have demonstrated that it is possible to learn multiple (conflicting) task constraints from physical practice alone, however, only when there is sufficient time for consolidation^62,63^.

Studies included in this review emphasized the importance for an appropriate visualization of error signals to trigger performance improvements. In most of the studies, observers viewed actors performing during their original practice sequences when learning to adapt to a certain perturbation (from initial high errors to subsequent lower errors). Additionally, it was shown that observation of high error trials (i.e., untrained practiced during early stages of learning) led to better learning gains as compared to observing low-error trials (i.e., trained practice trials during later stages of learning)^40^ Alternatively, visuomotor rotation paradigms^49–53^ also allowed to visualize error signals through an overlay of the actual movement trajectories of the hand and cursor trajectories. Adapted movements in a force-field may not be observable from an outside perspective if the movement trajectories are not displayed as compensatory movements and may only be found on the level of muscle forces. Therefore, it is important to observe the deviations resulting from a force field in an unlearned environment.

With respect to learning mechanisms, it has been suggested that the adaptation of internal models is triggered through the realization of task-errors and sensory prediction errors^24,64^. Task-errors reflect the discrepancy between required and achieved outcome following the completed action. The sensory prediction error, on the other hand, is a signal of the discrepancy between the expected and perceived sensory consequences of an action that is provided via feedback loops continuously during movement. Additionally, the presence or absence of aftereffects has been discussed as a marker of the successful implicit adaptation of forward models^24^. Previous research has shown that aftereffects are driven by sensory prediction errors^24^, but an increase or decrease of task errors can increase or decrease the strength of aftereffects^65,66^. Together, the fact that the observation of task errors is crucial for performance improvements but does not lead to aftereffects suggest that observational learning leads to a recalibration of inverse, but not forward models.

Studies in this systematic review also demonstrated a link between proprioceptive perception and observational learning. That is, on the one hand, observational^38^, similar to physical practice^67^, can alter the perceptual judgement of the position of the arm relative to the body midline after it was passively moved. This suggests that observational and physical practice both result in somatosensory change. In addition perceptual training prior to observational practice^45^, as well as passive movements to the correct target location in combination with observational practice^54^ facilitated learning. This suggests that perceptual training alters the ability to process and simulate the sensory signals of an observed action, leading to improved somatosensory representations. Findings of the involvement of proprioceptive perception in observational learning are also backed up by neurophysiological results of, McGregor, et al. ^43^, who found that blocking of the somatosensory cortex during action observation through magnetic stimulation of the median nerve negatively affected learning. These findings imply some contrasting evidence to the abovementioned results that the mechanisms of observational learning are explicit rather than implicit. Namely that despite the lack of actual sensory information from proprioceptors, observational learning can evoke changes in the somatosensory representation and that information processing in the somatosensory cortex plays a crucial role in observational learning.

Results of this review also indicate that observational in combination with physical practice has an advantage over isolated practice. While Ong, et al. ^53^ did not find any advantage in direct effects of mixed schedules over mere physical or observational practice, three other studies did in fact show beneficial effects^49–51^. They showed that (i) combined practice can facilitate learning of conflicting task constraints^49^; (ii) physical followed by observational practice of the same task-constraint led to better retention but reduced aftereffects^51^; (iii) and interspersed physical and observational practice compared to blocked schedules (with observational preceding or following physical practice) and pure physical or observational practice yielded best results for short- and long-term retention^50^.

The beneficial effect of combined observational and physical practice may be due to the integration of both, explicit and implicit learning strategies. Previous research has indeed shown that explicit and implicit processes are complementary learning strategies for physical practice. Particularly in the early phases of learning, explicit processes can lead to fast adaptations and implicit recalibration, resulting in more stable adaptations can follow later on^56^. However, Benson, et al. ^68^ have also pointed out that explicit instructions might also interfere and reduce implicit recalibration, leading to faster learning but less stable performance changes. Generally, the implicit adaptations are stronger and potentially also cancel out erratic explicit strategies^69^. While the advantage of combined over isolated practice might be due to complementary implicit and explicit learning mechanisms, further investigation is needed to identify whether a combined learning schedule has also negative effects, for example, regarding the stabililty of performance changes. Same applies for pure observational learning setups.

In sum, we highlight different factors that facilitate observational learning. First, the visualization of the task-error seems to be a key factor to demonstrate the nature of a perturbation or constraint to the observer. Second, observational learning appears to also include mechanisms of somatosensory information processing. Finally, combined practice schedules might foster complementary explicit and implicit learning mechanisms, leading to faster adaptation and reduced aftereffects^50^.

The underpinnings of observational learning have frequently been attributed to the mirror neuron system^10,11^, i.e., a system of brain areas that displays similar behaviour during the observation and execution of the same action^10,13,15^. Therefore, it is believed that mirror-like activation also leads to similar modification and consolidation of sensorimotor networks from observational as compared to physical practice^16,17^. In our literature review, we have identified only five studies that investigated the neurophysiological correlates of observational learning, using brain stimulation and neuroimaging. Most of these studies have focused on the involvement of M1 in observational learning^39,42–44,46^.

Interestingly, previous studies were ambiguous about a potential role of M1 in the mirror neuron system. Some studies in fact showed activation of M1 during action observation^70^. Yet, other studies showed inconsistent results^15^. Also on the cellular level, the existence of mirror neurons in the human M1 is being debated^71^. Hence, M1 neurons seem to display rather distinct activation patterns for action observation and execution. It is assumed that M1 activation during observation reflects an inhibitory, rather than a mirroring role^71,72^. Despite its ambiguous involvement in the mirror neuron system, M1 activity has been shown to modify adaptation effects following physical practice^24^. For example, an increase of the neuronal excitability of M1 (using anodal transcranial direct current stimulation) during physical practice increased retention and aftereffects following learning of a visuomotor rotation^73^ or force field^74^.

Evidence from fMRI studies further indicates the involvement of other structures including V5/MT, S1, PMd, SPL, as well as the cerebellum in observational learning ^42,44^Similar networks have been identified in studies on the neural correlates of motor learning through physical practice^24^, again underlining the strong overlap between both types of learning. For example, Vahdat, et al. ^75^ have found that physical practice of a force-field task led to changes in the functional connectivity between M1, PMd and the cerebellum. Further, studies on patients with cerebellar disorders have shown a reduced ability to adapt to novel task-constraints^76,77^. However, these networks can vary greatly depending on what is learned (e.g., visuomotor transformations, or novel skills) and how it is learned (e.g., associative, or procedural learning)^24^.

Overall, neural correlates of motor adaptation through observational practice appear to resemble those of adaptation through physical practice, such as the M1 and certain motor networks. However, evidence of neural correlates is sparse with only five studies in this review have investigated this, using a variety of different methodologies, and focusing on various areas. Given the explicit nature of observational learning, involvement of cognitive brain areas relevant for cognitive control, such as the dorsolateral prefrontal cortex^78^, is likely. So far, this has not yet been investigated. In addition, studies are lacking that directly compare the neural correlates of physical and observational practice in motor adaptation tasks. The notion that observational compared to physical practice has different effects on the behavioural level indeed suggests different brain mechanisms. Neurophysiological correlates do not yet provide conclusive evidence for this dissociation, however. Therefore, future studies should investigate the relationship between behavioural (aftereffects) and neural (cerebellar involvement) markers of observational practice to further unravel the role of forward models.

Surprisingly, evidence in this systematic review was based largely on the work of two research groups (Gribble and Hodges) which strongly limits the generalizability of the results. This is particularly since many of the individual findings and their interpretation (e.g., the absence of aftereffects) are either based on the findings of one group or the other. However, the paradigms used in both research groups seem to differ with respect to their underlying requirements to sensorimotor transformations. Understanding the nature of a velocity-dependent force-field requires integration of proprioceptive information whereas the nature of a visuomotor rotation might be easier to gauge. Thus, the use of explicit strategies might be, for example, much easier to counter a visuomotor rotation (simply aiming to a different location) as compared to a force-field. Only two studies^47,55^ were conducted in other research groups, out of which only one study used an entirely different paradigm^55^. Both groups addressed different research questions such as the absence of aftereffects or the neural correlates of observational practice.

This systematic review sought to bring results together and to discuss the effectiveness of observational practice for motor adaptation tasks with a special focus on aiming, and reaching studies. We found only reaching studies and all reaching paradigms in the included studies restricted the movement to two (translational) degrees of freedom. However, reaching usually encompasses three degrees of freedom. As such, future research may consider reaching tasks in a three-dimensional space. In addition, performance following observational practice was measured across studies through the largest deviation from a straight path. Other measures such as smoothness of movement^79^ may provide additional information about the quality of learning. Finally, to compare the brain mechanisms between physical and observational practice, it is desirable to include a physical and observational practice within one study. This has not been done yet.

Summing up the results, our systematic review demonstrates that observational like physical practice can lead to motor adaptations. However, both types of practice appear to evoke different learning mechanisms, as shown by the lack of aftereffects and better explicit task knowledge following observational practice. Therefore, it appears that physical practice rather evokes implicit and automatic adaptations whereas the mechanisms of observational practice are rather learning of explicit cognitive strategies. Neurophysiological studies, however, have demonstrated the involvement of different sensorimotor regions, such as the primary motor and somatosensory cortex, the premotor cortex as well as the cerebellum in observational learning. This indicates, at least to some extent, the involvement of implicit motor adaptations following observational practice. How and to what extent these implicit contributions might be up- or downregulated during observational practice^1^ remains to be explored. Due to the major limitation that some findings in this study (e.g., the absence of aftereffects) are predominantly based on only one of the used paradigms (force-field adaptations or visuomotor rotations), it is vital to additionally confirm and generalize individual findings in future studies.

Results of this systematic review may have strong implications for practice. In rehabilitation, for example following a stroke, functional impairments (such as spasticity) might arise in the upper extremities, posing constraints upon motor coordination. In such cases, it might be fruitful to learn and adapt from observing a model with a similar constraint physically practicing and successfully adapting, rather than a healthy model performing the task in a typical fashion. Additionally, observational practice might prove to be a suitable tool to enrich traditional types of learning (e.g., reinforcement learning) in robots. This systematic review has demonstrated some initial evidence about neural networks involved in observational learning of reaching which can inform the design of brain-inspired neural architectures that allow this type of learning in robots^2,57,80^.

## Methods

### Eligibility criteria

The inclusion criteria were defined by use of the PICO-model and the PRISMA guidelines for systematic reviews^81^. Studies were included in the review if they met the following criteria: (i) the mean age of the sample was 18 years and older, (ii) participants were healthy, (iii) observational learning was investigated by use of a reaching or aiming task, (iv) at least one pure (i.e., not mixed with physical practice) observational practice condition/group was included, (v) publications were written in English and (vi) the study was published in a peer-reviewed journal. Next, studies were excluded if they met the following criteria: if they investigated (i) learning of specific sequences (i.e., sequencing tasks), (ii) only motor learning without an observation condition/group, and (iii) if studies researched non-humans/animals. Studies that investigated observational motor practice with clinical samples were only considered as eligible when the study protocol comprised a healthy control group. Observational practice in reaching was considered if participants observed another person (e.g., an actor or another participant) performing a reaching task and if subsequently motor performance of the observer was tested. The year of publication was not considered as an eligibility criterion.

### Information sources

The systematic literature search was performed within the following four data bases: PsycInfo (coverage: 1806—present) using the EBSCO-host, PubMed (coverage: 1946—present), Scopus (coverage: 1788—present) and Web of Science (coverage: 1800—present).

### Search strategy

The original search was conducted on August 23rd, 2022, on abstracts, titles, and keywords. Four elements were combined using an AND operator. We used the following search-terms: (motor OR movement*) AND (learn* OR adapt* OR improve* OR acqui* OR memory OR consolid* OR training) AND (observ* OR immitat* OR mirror neuron*) AND (target task OR targeting OR grasp* OR reach* OR aiming task OR aiming) NOT (child* OR school* OR pupil* OR mice OR rat OR animal OR wetland* OR soil OR meta-analysis OR review). The considered papers had to be written in English language and published or accepted in a peer-reviewed journal.

### Selection process

After initial duplicate detection and deletion, two independent reviewers (L.H. and K.K.) screened the identified records chronologically by title, abstract and full text articles. Screening and duplicate detection and deletion was performed by using the software tool Rayyan for systematic reviews^82^. In case of disagreement between the two reviewers, a third (and fourth) reviewer (J.R., C.V.R.) was consulted to reach a consensus on the study selection. The search was complemented by use of a ‘snowballing’ system. That is, the reference lists of the original studies and additional scoping reviews that were identified in the search, were scanned for potentially relevant additional studies that were not identified in the primary search process.

### Data collection process

Data were extracted by two independent reviewers (L.H. and K.K.) for authors, title, and year of publication. Moreover, data was collected about the study design, type of task, descriptive data of the population (age, gender, handedness, sample size), reaching task, experimental condition, order of experimental conditions, task experience of the participants, presentation of observational stimulus, hand visibility, measurement device, test setup and outcome variables. Data was retrieved, controlled, and summarised in a table. Moreover, results were double checked and complemented by a third experienced researcher (JR). Discrepancies and disagreements in data extraction were resolved by discussion involving the entire author group.

### Data items

Performance parameters across the reaching tasks were: movement curvature/maximum perpendicular deviation (i.e., the maximum perpendicular distance of the reaching trajectories from a straight line between start and end point in mm); perpendicular displacement at peak speed (i.e., same as above but displacement was measured at the timepoint of maximum velocity, rather than maximum displacement in mm); directional error (directional distance between actual distance and a straight line between start and end point at the timepoint of maximum velocity in degrees). If outcomes were provided only graphically, we used Plot Digitizer (Arizona, US, Phoenix) to extract numeric results^83^.

Secondary outcomes were neural and behavioural correlates that reflect the brain mechanisms of observational learning in reaching tasks. These were extracted from studies using neuroimaging methods such as functional magnetic resonance imaging (fMRI) to analyse brain networks or non-invasive brain or peripheral stimulation (e.g., Transcranial Magnetic Stimulation) to measure corticomotor or somatosensory-cortical excitability. In addition, data on aftereffects of the above-mentioned measures of motor performance were sought as behavioural indicators of the mechanisms of motor learning.

Sample characteristics including the number of participants, age, gender, handedness, and country was extracted when available, to describe the samples and illustrate possible confounders and differences between studies. All available data on study designs, measurement setups and devices (e.g., robotic manipulandum or graphical tablet with visuomotor rotations), observation modalities, number of observations or practice trials, practice-, and test conditions (i.e., whether the post-test was congruent or incongruent to the training stimulus) were extracted to compare study results. Furthermore, the sequence of test and practice conditions, including the number of trials was extracted for each group in the different studies. To facilitate the comparability, terminology describing groups and conditions was aligned in our results where applicable. For example, if groups with the same test condition were reported, but under different names, they were unified (e.g., observation only (Obs_Only) to observation group (ObsG)). Also, test setup (subject position, start and target position, visual feedback) and instructions for the reaching tasks were extracted from the data and reported. When data of desired reach velocity and vision of the participant’s arm during reaching task were available, they were also listed.

### Study risk of bias assessment

The quality was assessed by using the revised Cochrane risk-of-bias tool (RoB2) using the recommendations described in chapter 8 of the Cochrane handbook for systematic reviews of interventions^84,85^. The RoB2 investigates the risk of bias in five domains: (1) Risk of bias arising from the randomisation process, (2) due to deviations from the intended interventions, (3) due to missing outcome data, (4) in measurement of outcome, (5) in selection of the reported result. Each domain was classified into high risk, some concerns and low risk of bias. The tool was applied to each of the included studies by two independent review authors (L.H. and J.R.). Any discrepancy between the resulting study qualities, was discussed after assessment and resolved by a third review author. The overall risk of bias judgement for every paper was deducted from the domains and summarised and classified as high risk (at least one of the domains was classified being at high risk of bias), some concerns (at least one of the domains was judged to raise some concerns, but none was at high risk), and low risk (all domains were considered low risk)^85^.

### Effect measures and synthesis methods

The individual studies were compared and structured with respect to testing and study design. Further, different control and intervention groups (see Table 2) were split into direct effects and aftereffects resulting from observational practice, physical practice or resting controls. For the direct effects, mean values and standard deviation were extracted from experimental groups that practiced stimuli that were congruent (perturbation in the same direction) or incongruent (perturbation in the opposite direction) to the test condition through observation, as well as control groups that observed an unlearnable stimulus or were naïve to the task. For the aftereffects, we extracted performance errors in an unperturbed condition following observational and physical practice. If studies only reported the standard error or confidence intervals as a measure of dispersion, it was converted to the standard deviation.

We used R (Version 4.2.2) and the package metafor^86^ for synthesis of the results. Standardized mean differences (Cohen’s *d*) were calculated for each study that reported a group mean as measure of dispersion. Cohen’s d was computed as the difference between mean values of each group divided by the pooled standard deviation: $d=\frac{{\bar{x}}_{1}-{\bar{x}}_{2}}{\sqrt{{s}_{1}^{2}-{s}_{2}^{2}}}$. We chose Cohen’s d due to different testing instruments and to make different kinds of reaching error comparable to each other. Effect sizes were compared using forest plots and random effects models were calculated to estimate the overall effects.

### Reporting bias and certainty assessment

If studies did not report all relevant measures (e.g., missing SD or SE) effect sizes could not be calculated. However, all reported measures were retrieved and displayed in the results section. In case the according effect size could not be calculated, the according cell in the table was left blank.

Certainty of the evidence was assessed and discussed by two independent reviewers (L.H. and R.J.) using the GRADE approach. The GRADE approach evaluates the evidence based on the following criteria: (1) Risk of Bias, (2) Inconsistency, (3) indirectness, (4) imprecision, (4) publication bias, (5) dose-response, and (6) effect size. Based on these considerations the certainty of evidence for important outcomes could be categorized into (1) high, (2) moderate, (3) low, or (4) very low.

## Supplementary information

Prisma Checklist

## Data Availability

All data generated and analysed in this manuscript can be accessed on Zenodo (10.5281/zenodo.12682543).

## Code availability

The code (R-Version 4.2.2 and package metafor) used to analyse the data can be accessed on Zenodo (10.5281/zenodo.12682543).
